# The Associations between Various Ectopic Visceral Adiposity and Body Surface Electrocardiographic Alterations: Potential Differences between Local and Remote Systemic Effects

**DOI:** 10.1371/journal.pone.0158300

**Published:** 2016-07-08

**Authors:** Po-Ching Chi, Shun-Chuan Chang, Chun-Ho Yun, Jen-Yuan Kuo, Chung-Lieh Hung, Charles Jia-Yin Hou, Chia-Yuan Liu, Fei-Shih Yang, Tung-Hsin Wu, Hiram G. Bezerra, Hung-I Yeh

**Affiliations:** 1 Division of Cardiology, Departments of Internal Medicine, Mackay Memorial Hospital, Taipei, Taiwan; 2 Medical Research, Mackay Memorial Hospital, Taipei, Taiwan; 3 Department of Medicine, Mackay Medical College, New Taipei City, Taiwan; 4 Mackay Medicine, Nursing and Management College, New Taipei City, Taiwan; 5 Department of Radiology, Mackay Memorial Hospital, Taipei, Taiwan; 6 Department of Gastroenterology, Mackay Memorial Hospital, Taipei, Taiwan; 7 Department of Biomedical Imaging and Radiological Sciences, National Yang Ming University, Taipei, Taiwan; 8 Cardiovascular MRI and CT Program, Baptist Cardiac Vascular Institute, Miami, United States of America; University of Bologna, ITALY

## Abstract

**Background:**

The associations between pericardial adiposity and altered atrial conduction had been demonstrated. However, data comparing differential effects of various body sites visceral adiposity on atrial and ventricular electrocardiographic alterations remains largely unknown.

**Methods and Results:**

We assessed both peri-cardial fat (PCF) and peri-aortic visceral adiposity (TAT) using dedicated computed tomography (CT) software (Aquarius 3D Workstation, TeraRecon, San Mateo, CA, USA), with anthropometrics including body mass index (BMI) and biochemical data obtained. We further related PCF and TAT data to standardized 12-leads electrocardiogram (ECG), including P and QRS wave morphologies. Among 3,087 study subjects (mean age, 49.6 years; 28% women), we observed a linear association among greater visceral adiposity burden, leftward deviation of P and QRS axes, longer PR interval and widened QRS duration (all p<0.001). These associations became attenuated after accounting for BMI and baseline clinical co-variates, with greater PCF remained independently associated with prolonged QRS duration (β = 0.91 [95% CI: 0.52, 1.31] per 1-SD increase in PCF, p<0.001). Finally, both PCF and TAT showed incremental value in identifying abnormally high PR interval (>200ms, likelihood-ratio: 33.17 to 41.4 & 39.03 for PCF and TAT) and widened QRS duration (>100ms, likelihood-ratio: 55.67 to 65.4 & 61.94 for PCF and TAT, all *X*^*2*^ p<0.05) when superimposed on age and BMI.

**Conclusion:**

We show in our data greater visceral fat burden may have differential associations on several body surface electrocardiographic parameters. Compared to remote adiposity, those surrounding the heart tissue demonstrated greater influences on altered cardiac activation or conduction, indicating a possible local biological effect.

## Introduction

Visceral adiposity, including the pericardial fat (PCF) and thoracic peri-aortic adipose tissue (TAT), has been shown to exert their biological functions which implicates in the pathogenesis of metabolic syndrome and atherosclerosis [1, 2]. Through its paracrine effects on adjacent myocardium [3], PCF is associated with metabolic derangements, insulin resistance and systemic inflammation [4, 5], together with certain cardiac structural and functional remodeling [6]. More recently, there is a growing body of literature has demonstrated that PCF may alter atrial electrical features, and the burden of PCF is tightly linked to atrial fibrillation (AF) incidence [7–9]. The underlying mechanisms were more likely to be associated with its detrimental effects in intra-atrial conduction [10] as the consequences of atrial remodeling.

On the other hand, TAT, the localized fat surrounding thoracic aorta remote from myocardium has also been shown to be associated with systemic inflammation [5] and some local cytokine secretions [11]. Recent studies indicated that TAT burden is related to increased aortic dimension [12] and altered vascular function [13]. However, whether this “remote” visceral adiposity may also exert some biological effects on cardiac electrophysiology remains poorly understood.

Based on these, we speculated that visceral adiposities may exert certain electrical remodeling on cardiac structures. While most prior studies focused on PCF and intra-atrial conduction indexes, little is known about various adiposity measures (either local or remote sites) on whole heart electrocardiographic characters, as featured by body surface electrocardiogram (ECG). The goal of the current work is to characterize these associations using computed tomography (CT) defined measures in an asymptomatic population.

## Materials and Methods

### Study population

From Jan 2005 through Dec 2012, a total of 3,411 consecutive subjects (mean age: 49.8±9.8) who participated in a primary cardiovascular health survey at a tertiary medical center in Taipei, Taiwan were enrolled. The settings, together with structured questionnaire, physical examinations, medical histories, detailed anthropometric measures and biochemical data were all obtained as previously published [5]. Patients with previous known cardiovascular diseases including prior myocardial infarction, stroke, coronary arterial disease, peripheral arterial disease or prior hospitalization for congestive heart failure were excluded. Among them, 3,194 had body surface ECG data available. Participants with baseline ECG conditions that may influence assessment of P wave morphology, PR interval or QRS-wave durations, such as advanced degree AV block, wandering atrial pacemaker rhythm, AF or flutter, pre-excitation, and any known form of bundle-branch block were all excluded.

The presence of a history of hypertension was defined as systolic blood pressure of more than140mmHg, diastolic blood pressure of more than 90mmHg, or previously diagnosed hypertension under pharmaceutical control. Diabetes mellitus was defined as fasting glucose level of more than126mg/dL or previously diagnosed diabetes mellitus under pharmaceutical control. Hyperlipidemia was defined a known history and/or the use of lipid lowering drugs, such as statins or fibrates, on a daily basis.

This study complies with the Declaration of Helsinki. It was approved by local ethical institutional committee (Mackay Memorial Hospital) for retrospective data analysis without informed consent of study participants (IRB No: 15MMHIS183e). Data security was guaranteed and all authors had no access to patient identifying information before and after data analysis. Study participants involved in this study were not under clinical service of current study physicians or researchers.

### Lab data acquisition and analysis

A Hitachi 7170 Automatic Analyzer (Hitachi Corporation, Hitachinaka Ibaraki, Japan) was used to measure fasting and postprandial glucose levels (hexokinase method), glycosylated hemoglobin, creatinine (kinetic colorimetric assay), total cholesterol and triglyceride. Lipid profiles including low-density lipoprotein and high-density lipoprotein cholesterol were obtained using homogenous enzymatic colorimetric assay. The estimated glomerular infiltration rate (eGFR) was derived from MDRD equation by the Chronic Kidney Disease Epidemiology Collaboration equation recommended by National Kidney Foundation [14].

### Multi-detector CT measurements of visceral adipose tissue quantification

Scanning was performed using a 16-slice multi-detector CT (MDCT) scanner (Sensation 16; Siemens Medical Solutions, Forchheim, Germany) with 16 * 0.75 mm collimation, rotation time of 420 msec, and tube voltage of 120 kV. In one breath hold, images were acquired from above the level of tracheal bifurcation to below the base of heart using prospective electrocardiographic triggering, with the center of the acquisition at 70% of the R-R interval. From the raw data, the images were reconstructed with standard kernel in 3 mm thick axial, non-overlapping slices and 25 cm field of view.

**Fig 1:**
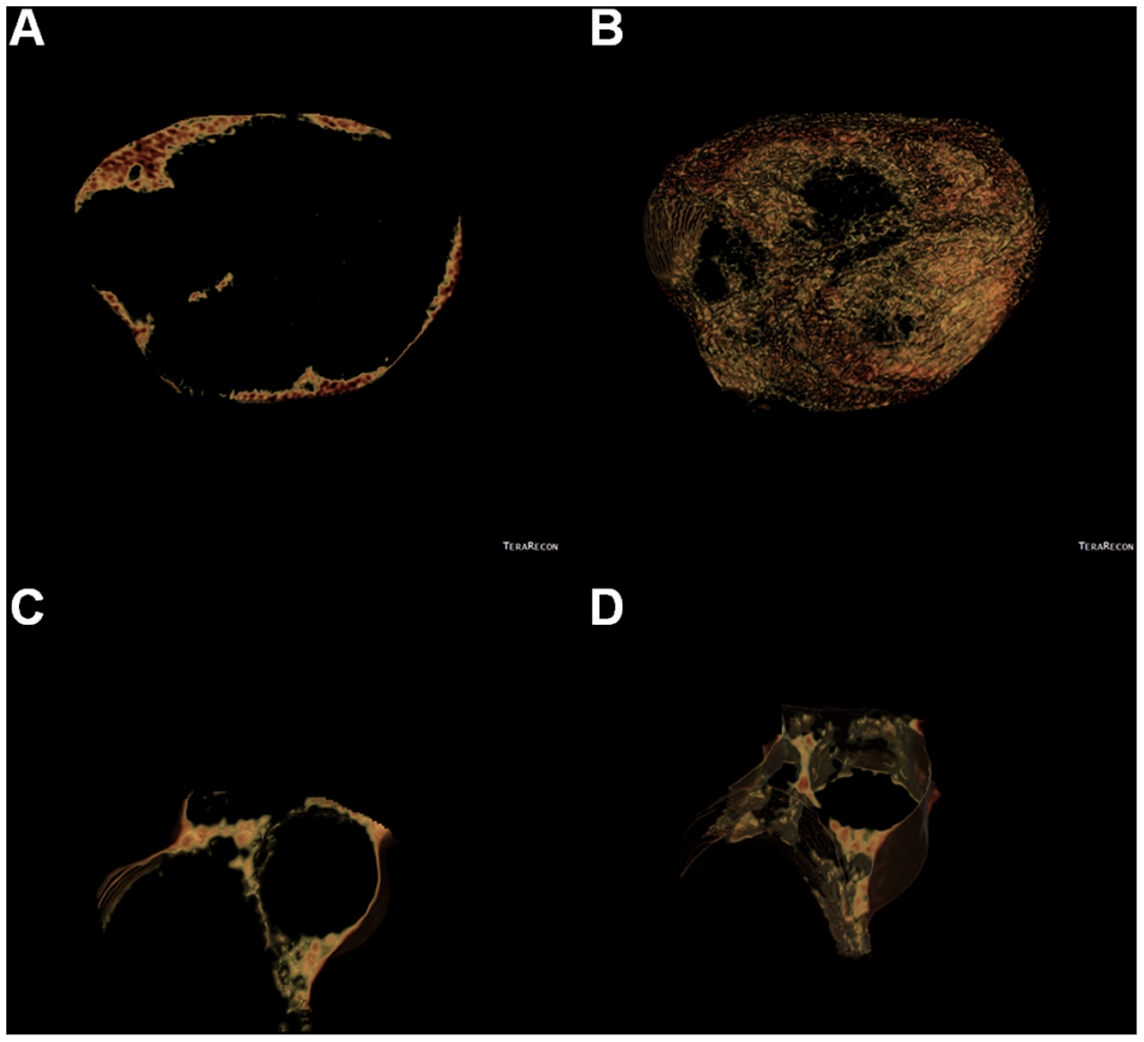
The illustration of single PCF (A) or TAT (C) slice re-construction by using dedicated CT software (Aquarius 3D Workstation, TeraRecon, San Mateo, CA, USA) and subsequent semi-automatic 3D volumes (B: PCF, D: TAT) using CT dataset by summation methods in our current study.

PCF volumes were quantified from the heart CT scan using a dedicated workstation (Aquarius 3D Workstation, TeraRecon, San Mateo, CA, USA). The semi-automatic technique was developed for quantification of adipose tissue volumes. We traced pericardium in axial MDCT images manually from the level of left main coronary artery to diaphragm every four to six slices. The computer software then automatically interpolated and traced pericardium along the manually traced areas. All automatically traced slices were verified and modified if necessary for accuracy. The definition of adipose tissue is pixels within a window of -195 to -45 HU and a window center of -120 HU. Peri-cardial fat measure (PCF) was defined as any adipose tissue located within the pericardial sac (Fig 1A and 1B), while peri-aortic adiposity (TAT) (Fig 1C and 1D) was defined by the area immediately surrounding the thoracic aorta (defined by a line drawn horizontally through the esophagus, which connected to the left costo-vertebral joint); and posteriorly by the right lateral border of the vertebral body and the anterior edge of the vertebral body. The pulmonary artery bifurcation served as the most rostral landmark and the area of interest extended caudally 67.5 mm. Of all 3194 subjects with 12-lead ECG data available, 3087 (96.6%) had complete CT data with good images quality rendered for visceral adiposity quantification. The intra-observer and inter-observer co-efficient variation of PCF and TAT were 4.27%, 4.87% as well as 6.58%, 6.81% in a random subset of 40 subjects [15].

### Body surface electrocardiogram

The resting 12-lead ECG was performed in all participants, using a Page WriterTC30 electrocardiograph (Phillip, Andover, USA) after a resting of period of at least 10 minutes. A standard ECG protocol was conducted with 10-second data stored for analysis. Examinations were recorded at a paper speed of 25 mm/s and an electric signal amplification of 1 mV/cm. Heart rate, P duration, PR interval, QRS duration, QT and QTc intervals, P-, QRS- and T-axes, P(II), S(V1), and R(V5) axes were all obtained and exported automatically from the recordings by PageWriter TC Cardiograph Software. The same software was used to detect the timings of all QRS complexes and calculated R-R intervals (ms) automatically. All ECGs strips were checked for falsely identified QRS complexes or non-sinus beats, and were further confirmed by physicians. Mean HR was calculated (in beats/min) as 60 divided by the mean R-R interval in seconds. The P and QRS axes were approximated by calculating the numerical sum of spatial deflections over time (amplitudes of positive deflections are added and those of negative deflections subtracted). PR abnormality or irregularity, other forms of cardiac rhythm anomaly including AF, flutter or wandering rhythm, and QRS morphology and patterns for bundle branch block were judged by two experienced cardiologists (CLH and PCC), together with implant of pacemaker by history taking and medical history review.

### Reproducibility of ECG measurements

Variability of ECG measurements in our department was assessed by 2 repeated ECGs recorded 1 week apart in a random sample of 30 subjects using same electrocardiograph equipment (Page WriterTC30, Phillip, Andover, USA), again with ECG-related parameters automatically derived from the recordings (PageWriter TC Cardiograph Software). The root mean squared difference between 2 ECG data for all relevant parameters were calculated and divided by the mean of the absolute values to represent each coefficient of variability (COV). The COV for P axis, PR interval, QRS axis, and QRS duration was 2.9%, 1.8%, 3.2%and 1.6%, respectively.

### Statistical analysis

Continuous data were presented as mean ± standard deviation [SD]. Continuous variables across graded subgroups were compared with nonparametric trend test (Wilcoxon-Rank-Sum test) across ordered groups, with categorical or proportional prevalence data expressed as proportion and compared by Chi square or Fisher Exact test as appropriate. The association of visceral adiposity (either PCF or TAT) as independent variable and various ECG indices as dependent variables was analyzed by linear regression, and further adjusted for key baseline clinical covariates, including age, sex, BMI, blood pressure, biochemical data (such as fasting sugar, lipid profiles), renal function in terms of eGFR, medical histories (such as hypertension, diabetes, hyperlipidemia, cardiovascular disease) and lifestyles (smoking and regular alcohol consumption) in multi-variate models. ECG indices including electrical axis, PR interval, QRS duration, and QT interval with and without corrections were sequentially entered into models. Owing to the collinearity, PCF and TAT were entered into models (as independent variables) separately, with linear regression or multi-variate models performed as mentioned above. The likelihood ratio test were used for comparisons of the incremental values of visceral adiposity (PCF or TAT) superimposed on age or BMI in identifying abnormally high PR interval (defined as PR >200ms) or longer QRS duration (defined as QRS >100ms).

All data was analyzed with the software STATA 9.0 package (Stata Corp., College Station, Texas). P value was set at two-tailed probability and a p value less than 0.05 was considered statistically significant.

## Result

### Baseline characteristics of study subjects

The baseline characteristics of the 3087 study subjects (mean age, 49.6 years; 28% women) are listed in Tables 1 and 2 according to PCF and TAT quintiles, with greater proportion of men than women across PCF and TAT quintiles. Individuals in the higher quintiles of PCF and TAT were older, with higher BMI, together with higher systolic and diastolic blood pressures (*p* for trend: <0.001). The associations between BMI, PCF and TAT were 0.5 & 0.55 (both p<0.001), with a positive linear association observed between PCF and TAT (r = 0.61, p<0.001). Greater fasting glucose, total cholesterol, LDL and TG level, together with lower HDL and eGFR in higher PCF and TAT groups were observed (*p* for trend: <0.001). Finally, individuals in the higher quintiles are more likely to have HTN, diabetes, hyperlipidemia, cardiovascular diseases and to be active smokers.

**Table 1:** Baseline demographic data of all study subjects based on visceral adiposity (either PCF or TAT) stratification in current study.

PCF, ml (25th -75th)	Q1: 34.04~45.94	Q2: 54.15~61.05	Q3: 67.59~74.95	Q4: 83.16~93.18	Q5: 105.75~134	p for trend
Age, years	45.25±9.31	48.6±8.83	49.71±9.02	50.95±9.5	53.66±9.89	<0.001
Gender, male (%)	321(10.40%)	429(13.90%)	468(15.16%)	498(16.13%)	521(16.88%)	<0.001
Weight, kg	59.44±10.23	65.11±11.37	68.98±10.72	71.73±12.46	76.77±12.32	<0.001
BMI, kg/m^2^	22.16±2.84	23.59±3.35	24.69±2.95	25.66±3.62	27.47±8.32	<0.001
SBP, mmHg	117.17±17.29	119.96±19.6	123.94±17.36	123.39±19.16	127.39±19.3	<0.001
WBC Count, X 10^3^/mm^3^	5.83±1.62	6.13±1.63	6.19±1.55	6.4±1.57	6.56±1.59	<0.001
Fasting Glucose, mg/dL	94.24±13.47	98.8±17.05	102.79±24.36	104.36±22.42	108.51±28.7	<0.001
Cholesterol, mg/dL	194.03±33.24	201.28±39.96	206.03±34.52	203.93±35.81	204.86±37.86	<0.001
Triglyceride, mg/dL	104.85±63.57	134.82±176.28	144.93±88.12	154.04±97.79	171.03±116.53	<0.001
LDL, mg/dL	121.18±30.03	129.94±31.94	134.81±31.17	133.82±32.74	134.06±33.36	<0.001
HDL, mg/dL	59.21±15.32	53.79±13.78	51.85±13.36	49.32±11.33	47.02±11.99	<0.001
eGFR, mL/min/1.73m^2^	86.69±15.69	82.7±14.19	81.48±13.67	81.36±15.41	79.95±15.65	<0.001
Hypertension, %	45(7.3%)	82(13.3%)	106(17.1%)	116(18.8%)	157(25.5%)	<0.001
Diabetes, %	13(2.1%)	35(5.7%)	30(4.9%)	39(6.3%)	56(9.1%)	<0.001
Hyperlipidemia, %	15(2.4%)	37(6%)	36(5.8%)	51(8.3%)	74(12%)	<0.001
CVD, %	12(2%)	20(3.3%)	20(3.2%)	36(5.8%)	46(7.5%)	<0.001
Alcohol use, %	29(4.7%)	30(4.9%)	35(5.7%)	43(7%)	43(7%)	0.241
Smoking, %	49(7.9%)	58(9.4%)	71(11.5%)	86(14%)	95(15.4%)	<0.001

Abbreviations: BMI: body mass index, CVD: cardiovascular diseases, eGFR: estimated glomerular filtration rate, HDL: high density apo-lipoprotein, LDL: low density apo-lipoprotein, PCF: pericardial fat, SBP: systolic blood pressure, TAT: peri-aortic adipose tissue, WBC: white blood cell count.

**Table 2:** Baseline demographic data of all study subjects based on visceral adiposity (PCF and TAT) stratification in current study.

TAT, ml (25th -75th)	Q1: 2.29~3.25	Q2: 4.14~5.07	Q3: 5.96~6.87	Q4: 7.82~9.14	Q5: 10.93~14.57	p for trend
Age, years	48.33±8.88	50.6±9.32	51.09±9	53.17±9.02	54.97±9.04	<0.001
Gender, male (%)	87(32.83%)	188(70.41%)	226(85.28%)	240(90.23%)	257(96.98%)	<0.001
Weight, kg	57.32±8.68	65.63±9.69	69.91±9.93	72.49±10.03	78.22±11.47	<0.001
BMI, kg/m^2^	21.89±2.76	23.73±2.63	24.91±2.98	25.68±2.94	27.35±3.38	<0.001
SBP, mmHg	115.11±16.52	121.73±16.89	123.9±15.54	127.36±14.89	128.71±16.31	<0.001
WBC Count, X 10^3^/mm^3^	5.62±1.49	6.01±1.55	6.22±1.58	6.55±1.57	6.72±1.63	<0.001
Fasting Glucose, mg/dL	93.14±11.26	98.03±16.05	101.81±22.83	102.7±20	112.99±31.43	<0.001
Cholesterol, mg/dL	195.77±35.08	201.89±35.54	204.3±36.46	204.88±38.71	203.24±36.32	<0.001
Triglyceride, mg/dL	94.54±55.62	125.4±72.15	142.73±86.89	162.99±178.58	183.95±126.25	<0.001
LDL, mg/dL	121.2±31.32	130.09±32.23	135.13±32.31	135.35±30.82	132.28±32.5	<0.001
HDL, mg/dL	62.37±15.43	55.22±13.57	50.69±12.18	47.44±10.01	45.4±10.2	<0.001
eGFR, mL/min/1.73m^2^	87.4±15.06	84.39±15.56	82.03±13.92	80.62±13.86	77.7±15.28	<0.001
HTN, %	38(6.1%)	67(10.9%)	83(13.5%)	115(18.6%)	203(33%)	<0.001
DM, %	12(1.9%)	20(3.3%)	35(5.7%)	36(5.8%)	70(11.4%)	<0.001
Hyperlipidemia, %	15(2.4%)	32(5.2%)	46(7.5%)	50(8.1%)	70(11.4%)	<0.001
CVD, %	11(1.8%)	29(4.7%)	24(3.9%)	23(3.7%)	47(7.6%)	<0.001
Alcohol use, %	21(3.4%)	26(4.2%)	33(5.4%)	44(7.1%)	56(9.1%)	<0.001
Smoking, %	31(5%)	54(8.8%)	72(11.7%)	96(15.5%)	106(17.2%)	<0.001

### The association between visceral adiposity and body surface ECG parameters

**Fig 2:**
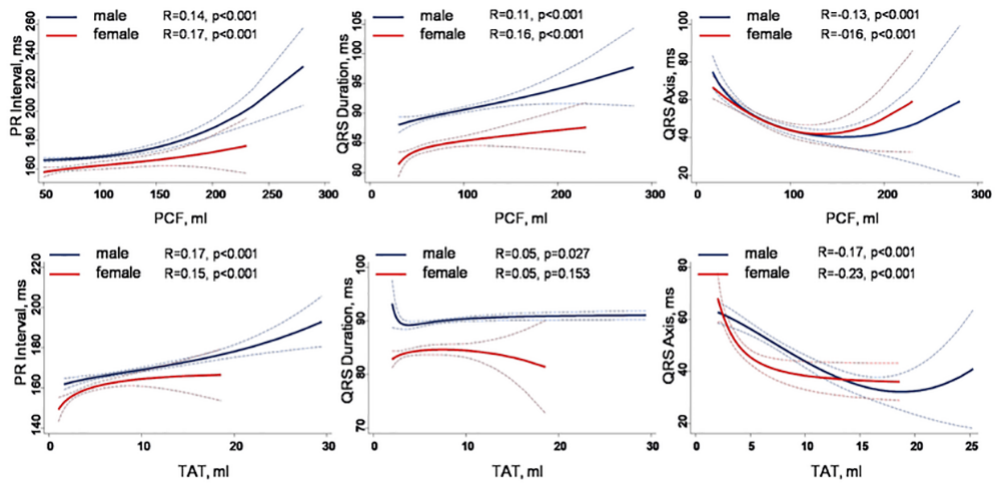
The associations between PCF or TAT on several electrocardiographic indices, including PR interval, QRS duration and QRS axes. No sex interactions were observed n these associations.

As shown in Table 3, the axes of the P, QRS and T waves were progressively left-shifted with greater volume of PCF and TAT (all *p* for trend < 0.001). The PR interval and QRS duration were significantly longer in higher quintiles of PCF and TAT (*p* for trend < 0.001). A positive linear association between visceral adipose burden (either PCF or TAT) and PR interval, QRS duration and axis were further shown in Fig 2. In unadjusted linear regressions (Table 4), both greater PCF and TAT volumes (per 50ml and 5 ml increase, respectively) were associated with more leftward axes of P, QRS and T waves. Per 50ml increase of PCF is associated with 6.2ms increase in PR interval, nearly 2.5ms increase of QRS duration, and a leftward QRS axis deviation of 8.9degree (all p < 0.001), with per 5ml increase of TAT is associated with 5.8ms increase in PR interval, 2.1ms increase of QRS duration, and a leftward QRS axis deviation of 8.96 degree (all p < 0.001). Both PCF and TAT increase were related to leftward P axes deviation (6.5 & 4.5 degree per 1 SD increase of PCF & TAT). For all relationships between visceral adiposity (either PCF or TAT) and various ECG parameters as dependent variables, there were no sex modification effects (all sex interactions p = NS).

**Table 3:** Several body surface 12-lead ECG parameters across visceral adiposity stratification measures (PCF and TAT) in current study

**PCF, ml**	**Q1: 34.04~45.94**	**Q2: 54.15~61.05**	**Q3: 67.59~74.95**	**Q4: 83.16~93.18**	**Q5: 105.75~134**	**p for trend**
P Axis,°	54.77±24.38	52.01±22.5	48.49±25.78	45.56±25.87	43.76±23.38	<0.001
PR Interval, ms	160.38±19.89	163.95±18.99	164.97±20.29	166.54±20.04	170.69±20.89	<0.001
QRS Axis,°	58.78±34.51	52.58±38	51.13±37.6	44.21±39.17	42.04±42.9	<0.001
QRS Duration, ms	85.65±8.89	87.58±8.3	88.89±8.8	88.98±8.53	90.58±8.52	<0.001
**TAT, ml**	**Q1: 2.29~3.25**	**Q2: 4.14~5.07**	**Q3: 5.96~6.87**	**Q4: 7.82~9.14**	**Q5: 10.93~14.57**	**p for trend**
P Axis,°	52.73±26.59	51.53±25.12	50.69±23.97	46.51±24.52	43.25±22.04	<0.001
PR Interval, ms	158.08±18.68	163.33±19.84	165.08±19.97	168.57±18.8	171.38±21.54	<0.001
QRS Axis,°	58.48±33.52	53.46±38.5	51.78±36.06	46.21±39.3	38.84±43.92	<0.001
QRS Duration, ms	85.14±8.86	88±8.36	88.34±8.62	89.96±8.48	90.2±8.53	<0.001

**Table 4:** The associations between visceral adiposity and body surface ECG data in uni-variate and multi-variate models

**Uni-variate**	**PCF (per +50ml)**			**TAT (per +5ml)**			**Multi-variate**	**PCF (per +50ml)**			**TAT (per +5ml)**		
	Coef	SE	p value	Coef	SE	p value	**Model 1**	Coef	SE	p value	Coef	SE	p value
P Axis,°	-6.53	0.70	<0.001	-4.54	0.54	<0.001	P Axis,°	-7.85	0.76	<0.001	-6.82	0.65	<0.001
PR Interval, ms	6.22	0.58	<0.001	5.84	0.44	<0.001	PR Interval, ms	3.25	0.61	<0.001	2.90	0.52	<0.001
QRS Axis,°	-8.94	1.05	<0.001	-8.96	0.81	<0.001	QRS Axis,°	-6.21	1.14	<0.001	-7.91	0.97	<0.001
QRS Duration, ms	2.52	0.32	<0.001	2.06	0.24	<0.001	QRS Duration, ms	1.71	0.34	<0.001	0.65	0.29	0.025
**Multi-variate**	**PCF (per +50ml)**			**TAT (per +5ml)**			**Multi-variate**	**PCF (per +50ml)**			**TAT (per +5ml)**		
**Model 2**	Coef	SE	p value	Coef	SE	p value	**Model 3**	Coef	SE	p value	Coef	SE	p value
P Axis,°	-5.05	0.81	<0.001†	-4.23	0.70	<0.001†	P Axis,°	-4.48	0.87	<0.001†	-3.54	0.77	<0.001†
PR Interval, ms	2.04	0.66	0.002†	1.79	0.57	0.002†	PR Interval, ms	1.80	0.71	0.011†	1.3	0.63	0.037†
QRS Axis,°	-3.82	1.22	0.002†	-6.08	1.06	<0.001†	QRS Axis,°	-3.04	1.29	0.019†	-4.72	1.16	<0.001†
QRS Duration, ms	1.64	0.33	<0.001†	0.43	0.29	0.132†	QRS Duration, ms	1.69	0.36	<0.001	0.36	0.27	0.171

Model 1: adjusted for age, gender;

Model 2: adjusted for age, gender, BMI;

Model 3: adjusted for age, gender, BMI, SBP, Pulse rate, HDL, LDL, eGFR, hypertension, diabetes, cardiovascular disease, current smoking and alcohol use.

^†^ in which models BMI was significant (p<0.05).

**Fig 3:**
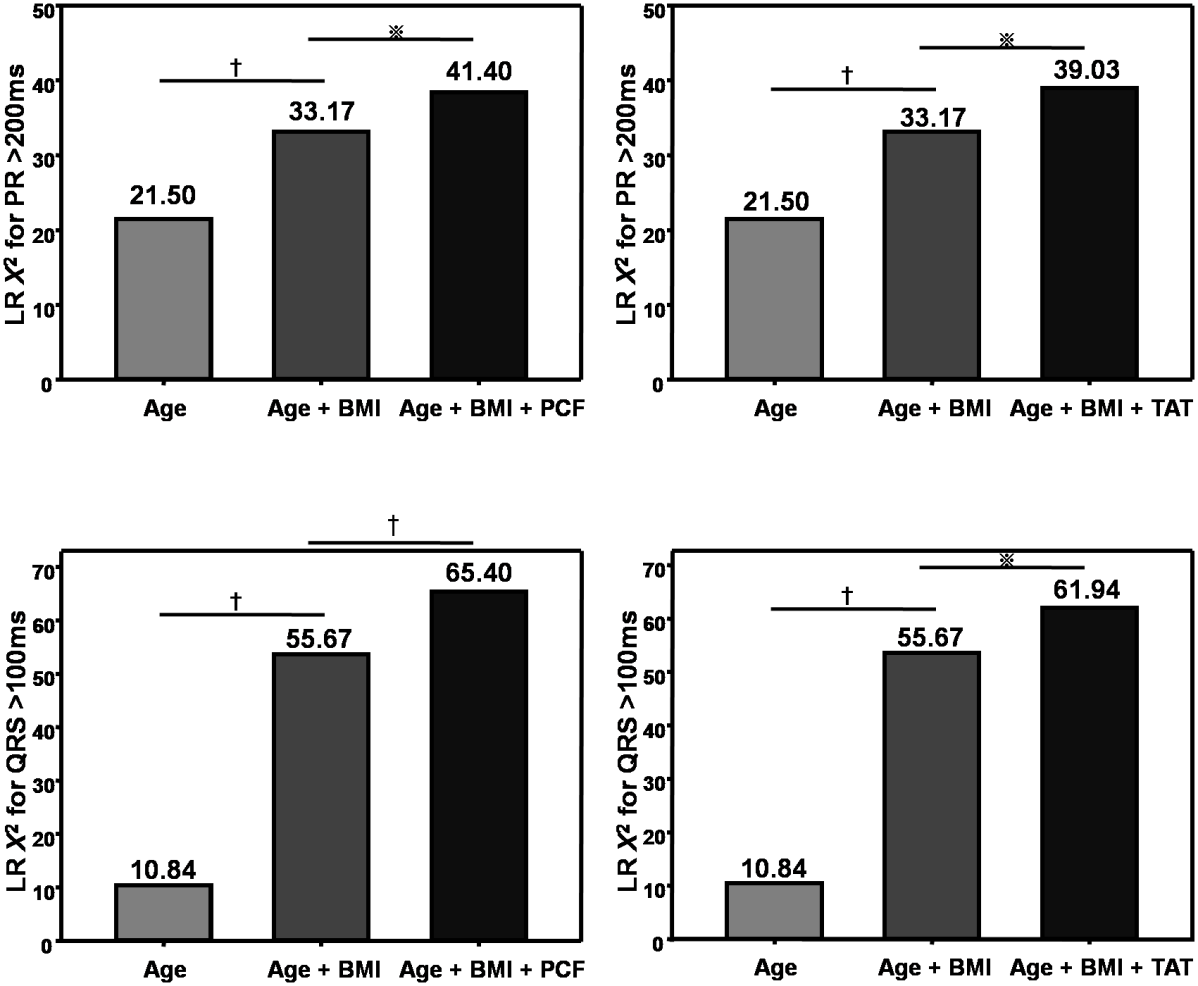
Likelihood ratio tests demonstrated the incremental value of PCF and TAT in identifying body surface 12-leads ECG anomaly, including prolonged PR interval (>200ms) and QRS duration (>100ms) when superimposed on age and body size measure in terms of BMI.

PCF and TAT above 75% upper limit (corresponding to 93.19, 9.21ml) in our population are associated with OR: 1.58, 95% CI: 1.14–2.2, p = 0.006 & 2.4, 95% CI: 1.43–4.04, p = 0.001 for abnormally higher PR interval (> = 200ms), and associated with OR: 1.62, 95% CI: 1.26–2.09, p<0.001 & 1.46, 95% CI: 1.13–1.89, p = 0.004 for abnormally higher QRS duration (> = 100ms). Compared to PCF at 5% lower limit, those with PCF at 95% upper limit showed 16ms longer PR interval, and 6.7ms longer QRS duration. Further, those with TAT at 95% upper limit demonstrated 21.8ms longer PR interval, and 6.0 ms longer QRS duration compared to those at 5% lower limit in our population. When PCF and TAT were separately superimposed on age and BMI, there are significant increase of chi-square value in identifying abnormally higher PR interval (>200ms) or QRS duration (>100ms) using likelihood-ratio test (Fig 3, *X*^*2*^ values: from 33.17 to 41.4 & 39.03 for PCF and TAT in predicting abnormally high PR interval [>200ms]; from 55.67 to 65.4 & 61.94 for PCF and TAT in predicting abnormally high QRS duration [>100ms], respectively, all p<0.05).

#### Multi-variate analysis

After adjustment for age and gender (Table 4, model 1), the independent associations between increased TAT or PCF burden (per 50ml and 5 ml increase, respectively) and all ECG variables remain unchanged (all p<0.05), though the association between TAT and QRS duration becomes attenuated after further adjustment for BMI (Coef: 0.43, p = 0.NS in Table 4, model 2). After further adjusting for other clinical co-variates related to cardiovascular diseases, both increased PCF and TAT burden are still associated with leftward P (Coef: -4.48 and -3.54), longer PR internal (Coef: 1.8 and 1.3), and leftward QRS axes deviation (-3.04 and -4.72, respectively, all p<0.05. Table 4, model 3), though QRS duration only remains significantly associated with greater PCF (Coef: 1.69, p<0.001) rather than TAT (Coef: 0.36, p = NS). BMI was also statistically significant in all relevant multi-variate models (with electrophysiological features as dependent variable) except for QRS duration. Compared to TAT, PCF demonstrated greater effects on all these associations.

## Discussion

In this study, we investigated the associations between visceral adiposity measures, either PCF or TAT, and several body surface electrocardiographic features in a large Asian population of 3,087 subjects. The main findings were 2-folds: 1) both PCF and TAT volumes were negatively associated with P and QRS-wave axes and positively associated with PR interval. These associations remained independent even after accounting for clinical variables that potentially may produce atherosclerosis and myocardial hypertrophy; 2) greater PCF was strongly associated with longer QRS duration, with TAT showed modest correlation which attenuated after adjustment.

### PCF, TAT and LA remodeling

Greater visceral adiposity burden, including both PCF and TAT, had been proposed associated with systemic inflammation, metabolic derangements, and a variety of cardiovascular diseases [1, 2, 15–17]. Even though, their biological roles and behaviors may vary to a degree. For example, PCF has been shown to exert local inflammatory effects that correlate inflammatory cell infiltrates within PCF [18], and shows higher pro-inflammatory cytokines (eg. resistin) or lower anti-inflammatory cytokine (eg. adiponectin) when compared to gluteal adipose tissue [19]. Instead, leptin expression was significantly higher in peri-aortic adipose tissue [11]. On one hand, given the lack of fibrous, fascial layer of myocardium to isolate free fatty acids and adipocytokines from surrounding adipose tissue, PCF may influence the myocardium directly through epicardial to subepicardial connective tissue between the muscle bundles [20]. On the other hand, a remote, systemic biological inflammatory effect had also been reported in certain visceral adipose tissue [19, 21].

Owing to the relatively thin wall and abundant epicardial adiposity, the local effects of PCF on atrial remodeling may be theoretically more pronounced [16, 17]. Further, increased amount of PCF may directly cause impaired diastolic filling through its mechanical compression to prevent ventricle from elastic expansion, resulting in subsequent atrial enlargement [22]. On the contrary, it has been shown that TAT volume was associated with aortic dimension and certain vasculopathy [12, 13] and major adverse cardiovascular events [23]. Even though, little is known for the potential remote relationship between TAT and human atrial remodeling.

### PCF, TAT and cardiac electrical remodeling: associations with PR interval

Though mainly accumulated in the atrioventricular (AV) and interventricular grooves along coronary arteries, PCF also encompasses several cardiac structures, including the atria, the apex of the left ventricle and right ventricular free wall [24]. In addition, much of this overlays the specialized conduction system responsible for atria conduction in between the SA and AV node given the anatomic proximity [25] which may serve as routes for most efferent parasympathetic nerve fibers [26]. Therefore, the expansion of such adipose tissue may produce certain pathological effects including inflammatory cytokines on cardiac conduction, especially those obesity individuals [20, 22, 24]. Among several electrocardiographic parameters, prolonged PR interval had been proposed a marker of atrial structural or electrical remodeling which likely reflect intra- or inter-atrial conduction delay between SA and AV node [27], and had been shown to be associated with heart failure or AF initiation and perpetuation in several longitudinal studies [28–30].

Consistent with our findings, prior epidemiological reports from Framingham and MESA study had demonstrated the associations between PCF burden and atrial conduction anomaly [31, 32]. However, in our multivariable analysis we showed that both PCF and TAT are independently associated with prolonged PR interval even after further adjusting for age and sex, though these associations became attenuated after further adjustment for BMI and other traditional cardiovascular risks. In addition, both excessive PCF and TAT added significant incremental values in identifying abnormally long PR interval beyond BMI. While obesity had been recognized to be associated with atrial electrical remodeling (such as widening P wave or prolonged PR interval) and a major risk for AF through local or systemic influences [33], the independent link between larger PCF/TAT and PR prolongation beyond BMI further support the possible role of excessive visceral adiposity in AF substrate formation [7–10]. Nevertheless, the possible mechanisms underlying these relationships may be somewhat different between PCF and TAT, with PCF presumably mediates its role through local effect owing to its unique anatomy exclusively encased within pericardial sac adjacent to myocardium [31, 34].

### PCF, TAT and cardiac electrical remodeling: associations with QRS duration

We also observed that certain ventricular activation patterns by electrocardiogram (QRS-wave axis and duration) were influenced with accumulated adiposity in our work. In addition to the possible displacement of the heart by an elevated diaphragm [35] in subjects with increased visceral fat burden, enriched myocardial triglyceride content is related to frontal plane QRS axis deviation in subjects metabolic derangements [36], which may reflect the degree of abnormal fatty acid infiltration in myocardium causing certain functional disturbance. Left ventricular hypertrophy (LVH) as a consequence to excessive body size accompanied by abundant visceral adiposity depots [37] or relevant to any obesity-induced cardiac structural abnormalities [38] may underlie intra-ventricular conduction or functional disturbances, leading to wider QRS duration. Excessive myocardial lipid infiltration and impaired O_2_ delivery from ventricular hypertrophy may also cause altered calcium handling or elevated oxidative stress from reactive oxygen species, resulting in abnormal cardiomyocyte excitation-contraction coupling [39, 40]. Widened QRS duration in individuals with obesity may likely reflect altered ventricular conduction due to underlying diseases and elevated risk of sudden cardiac deaths [41]. Interestingly, we noticed that BMI effects were attenuated in multi-variate models with both PCF and TAT remained significant. These data suggested that association of obesity on QRS duration may be better explained by biological visceral adiposity accumulation and related effects rather than BMI *per se*. Further, the strong and independent association between widened QRS duration and greater PCF in multi-variate models rather than TAT suggested a more direct mechanistic link between local visceral depots (e.g. PCF) and QRS widening, rather than via remote or systemic effect (e.g. TAT). Even though, the possible distinct mechanisms as mentioned above may need to be clarified in future studies. Taken together, elevated myocardial fibrosis and increased lipid content in the heart may eventually lead to slowed ventricular conduction [42], generating cardiac electromechanical dyssynchrony [43]. Indeed, we show in our data that these effects may be independent from traditional body size measures, with PCF remains more tightly adhered to QRS duration when compared to TAT.

## Limitations

The study has multiple limitations. First, our cohort included fewer male than female, which may limit its generalizability. Secondly, we did not include the data of heart chambers or ventricular mass, which might be helpful to further elucidate whether the observed ECG changes may be in part driven by these cardiac structural alterations. Thirdly, we would like to emphasize that the observed small to modest change of ECG parameters relevant to accumulating visceral adiposity should be interpreted with caution. Owing to the fact that more obvious ECG alteration may tend to happen at much higher level of adiposity accumulation and that a considerable amounts of the reported range of visceral adiposity (PCF and TAT) were associated with a variation of these ECG parameters (e.g. PR and QRS, QRS axis) within the reported values of COV (Coefficient of variability) in visceral adiposity measures, the real clinical impact of the analyzed variables is limited. Finally, the cross-sectional nature of the study precludes determination of the temporal relation or causality regarding the development of PCF burden and changes in ECG variables. Future longitudinal studies or interventions to reduce visceral adipose tissue burden in this population may help to clarify these relationships.

## Conclusion

PCF and TAT, which likely represent different body site visceral adiposity, seem to correlate several body surface electrocardiographic alterations with diverse results. These data may be interpreted as their differential biological behaviors. While both PCF and TAT are related to prolonged atrio-ventricular conduction, the association between intra-ventricular conduction delay was more pronouncedly produced by PCF, which is likely to be explained by its local biological role via direct contact with myocardium and functional proximity. Instead, TAT may mediate relevant influences via alternative pathways, possibly by remote or systemic mechanism.
